# Iron-related dietary pattern increases the risk of poor cognition

**DOI:** 10.1186/s12937-019-0476-9

**Published:** 2019-08-29

**Authors:** Zumin Shi, Tahra El-Obeid, Ming Li, Xiaoyue Xu, Jianghong Liu

**Affiliations:** 1 Human Nutrition Department, College of Health Science, QU Health, Qatar University, Doha, Qatar; 2 Centre for Population Health Research, Division of Health Sciences, University of South Australia, Adelaide, Australia; 3 Faculty of Health, University of Technology Sydney, Sydney, Australia; 4 University of Pennsylvania School of Nursing, Philadelphia, USA

**Keywords:** Cognitive function, Dietary pattern, Lead intake, Chinese, Adults

## Abstract

**Introduction:**

High iron intake has been shown to be associated with poor cognition. We aimed to examine the association between iron-related dietary pattern (IDP) and cognitive function in Chinese adults.

**Method:**

Longitudinal study data from the China Health and Nutrition Survey (CHNS) during 1991–2006 were used (*N* = 4852, ≥55 years old). Dietary intake was obtained from a 3-day food record during home visits. Reduced rank regression was used to construct IDP with iron intake as a response variable. Cognitive function was assessed in 1997, 2000, 2004 and 2006. Multivariable mixed linear regression and logistic regression were used in the analyses.

**Results:**

IDP was characterised by high intake of fresh vegetable, wheat, legume, beverage, offal, rice and whole grain. High IDP intake was associated with poor cognition. In fully adjusted models, across the quartiles of IDP, the odds ratio (95% CI) for poor cognitive function were: 1.00, 1.06 (0.86–1.30), 1.24 (0.99–1.54), and 1.50 (1.17–1.93), respectively. There was a borderline significant interaction between IDP and meat intake (p interaction 0.085). The association between high IDP and poor cognition was only observed among those with no or low intake of meat. With the adjustment of carbohydrate or iron intake, the IDP and cognition association became non-significant. IDP was positively associated with lead intake. The association between IDP and poor cognition was partly mediated by lead intake.

**Conclusions:**

Iron-related dietary pattern is associated with poor cognition in Chinese adults, partly due to high intake of carbohydrate, iron and lead.

**Electronic supplementary material:**

The online version of this article (10.1186/s12937-019-0476-9) contains supplementary material, which is available to authorized users.

## Introduction

Worldwide, dementia was estimated to affect 35.6 million people in 2010, and this number is expected to reach 115.4 million by 2050 [1]. It affects approximately 9.5 million adults aged 60 years and above in China [2]. As there is still no effective treatment to delay the progression of dementia, identifying modifiable risk factors other than hypertension, diabetes and stroke that can be used in the early prevention is urgently needed. The role of diet on cognitive decline and dementia has increasingly attracted attention [3, 4].

Many nutrients (e.g. fiber, carbohydrate, protein, docosahexaenoic acid (DHA)) and foods (e.g. alcohol, nuts, fish) have been assessed for their potential effects on cognition [4]. The traditional single nutrient and disease association approach does not consider the possible synergy or interaction between nutrients. The use of dietary pattern using either a priori (e.g. healthy eating index, Mediterranean diet score) or a posterior (e.g. factor analysis, cluster analysis) approach is becoming popular in nutritional epidemiology [5]. While a high proportion of studies uses factor analysis to construct dietary pattern, there is also an increasing number of studies using Reduced Rank Regression (RRR) to construct dietary pattern [6]. The advantage of RRR method is that it uses biomarkers or nutrients as intermedia responses so that it can explore the potential mediation effects of diet on disease [6].

Similar to the studies in Western countries [7], several studies in Asia suggested a link between dietary patterns and cognition among older adults. For example, in Korea, using RRR method with responses regarding vitamin B6, vitamin C, and iron intakes, a dietary pattern characterized by high intake of seafood, vegetables, fruits, bread, snacks, soy products, beans, chicken, pork, ham, egg, and milk was found to be associated with a decreased risk of mild cognitive impairment [8]. A diet with high intakes of vegetables, soy products, fruit, and fish may have a beneficial effect on cognitive function in older Japanese people using the similar approach [9].

There is increasing evidence from epidemiological studies suggesting the link between high iron levels and chronic diseases including dementia. Basic science also demonstrates that iron accumulation in the brain increases with age [10]. In the Western countries, higher dietary iron intake has been found to be associated with higher risk for Parkinson disease [11, 12]. In many countries, it has been shown that high iron levels increases the risk for diabetes [13], which is linked to cognitive impairment [14]. Despite these initial findings, the association between iron intake and cognition among older adults is inconsistent, as both positive [15, 16] and negative [17] associations have been reported.

Additionally, studies have shown that the modern Western diet with higher simple carbohydrates and saturated fat intake is correlated with cognitive impairment [18]. Conversely, clinical intervention studies of very low carbohydrate (5–10% of total calories) consumption shows improved verbal memory in the elderly [19] as well as improved overall cognitive performance in adults with type 2 diabetes [20, 21].

Furthermore, cognitive impairment has been found to be associated with both occupational and non-occupational lead exposure as indicated by blood and bone lead [22]. In the general population, diet is a major source of heavy metals in human body [23]. However, the number of studies on the association between dietary pattern and heavy metal contamination is limited. Lead levels have been implicated in impaired cognitive function across the lifespan [24]. It is unknown whether the association between dietary pattern and cognitive impairment is mediated by heavy metal contamination. We have recently reported a positive association between high iron intake and poor cognition among Chinese adults [17]. However, given the possible interaction effects between nutrients, it is important to look at overall dietary pattern rather than a single nutrient. Iron intake could be marker of other unmeasured factors. In China, the major source of dietary iron is from plant-based food. At the same time, lead contamination on plant-based food is of concern. For example, data from a large surveillance system in China suggested that 6.4% of cereal grain and pulse samples had lead concentration exceeding the maximum level of 0.2 mg/kg [25]. Lead intake has been found to be positively associated with all-cause mortality [26]. Plant-based food is also the major source of carbohydrate. Furthermore, exposure to lead has been consistently associated with cognitive impairment across the lifespan [27, 28] and has been demonstrated to reduce cognitive function in older adults [29, 30]. Thus, in the current study, we aimed to assess the iron related dietary pattern using RRR method and cognition among Chinese adults attending China Health and Nutrition Survey (CHNS). The second aim of the study was to test whether the association between the dietary pattern and cognition was mediated by lead or carbohydrate intake.

## Methods

### Study design and study sample

This study used repeated measurements of dietary intake and cognitive function over 15 years since 1991, from the CHNS [31, 32].The CHNS study is an ongoing open prospective household-based cohort study conducted in nine provinces covering both urban and rural areas spanning across Northern to Southern China. Nine waves of data collection (i.e. 1989, 1991, 1993, 1997, 2000, 2004, 2006, 2009, and 2011) have been conducted. Cognitive screen tests were conducted among those above age 55 years in 1997, 2000, 2004 and 2006 surveys. In total, 4852 participants attended the cognitive screen tests between 1997 and 2006 (Additional file 1: Figure S1). Participants who completed at least one cognitive screen test were included in the analysis. Of these participants, 3302 attended the screen test in at least two surveys. The survey was approved by the institutional review committees of the University of North Carolina (USA) and the National Institute of Nutrition and Food Safety (China). Informed consent was obtained from all participants. The response rate based on those who participated in 1989 and remained in the 2006 survey was > 60%.

### Outcome variable: cognitive function

The cognitive screening items used in CHNS included a subset of items from the Telephone Interview for Cognitive Status–modified [33]. The screening test was conducted face-to-face during home visit. The screening included immediate and delayed recall of a 10-word list (score 10 for each), counting backward from 20 (score 2), and serial 7 subtraction (score 5). A total verbal memory score was calculated as the sum of the immediate and delayed 10-word recall. The total global cognitive score ranges from 0 to 27. A high cognitive score represents a better cognition. The cognitive function test started with the immediate recall of a 10-word list. The interviewer (i.e. trained health worker) read ten words at a speed of 2 s per word. The participants were given 2 min to memorize the ten words. For each correct recalled word, a score of 1 was given. The participants were then asked to count back from 20 to 1. If the participants made a mistake in the first try, a second chance was given. A score of 2 was given to those answered correctly in the first try, or 1 in the second try. After the count test, the participants were asked to do five consecutive subtractions of 7 from 100. Each correct subtraction was given a score of 1. Finally, the participants were asked to recall the 10-word list tested before. Each recalled word was given a score of 1. In our study, we choose the first quintile of the cognitive function test score as poor cognitive function, which corresponds to a global cognitive function score cut off of < 7. The cutoff was selected based on a study in Shanghai which showed that the prevalence of mild cognitive impairment among people aged 60 and above was 20% [34].

### Exposure variables: Iron related dietary pattern (IDP) and lead

#### Iron related dietary pattern

Individual food intake was recorded on three consecutive days by a trained investigator at each wave. In the 3-day dietary survey, foods and condiments in the home inventory, foods purchased from markets or picked from gardens, and food waste were weighed and recorded. Nutrients intakes including iron and carbohydrate were calculated based on the average of 3-day food consumption data using the Chinese Food Composition Table [35] . The dietary assessment method has been validated for energy intake [36]. Based on similar nutrient profiles or culinary use, food intake were collapsed into 35 food groups, and average food intake for individuals (gram/day) calculated for each wave. Soft drinks, fruit juice and tea are categorised as beverage. The food groups are similar to the food items used in a validated food frequency questionnaire used in a 2002 Chinese national nutrition survey. The detailed description of the dietary measurement has been provided in the previous publication [31].

IDP was constructed using RRR analysis with the intakes of 35-collapsed food groups as input variables. PROC PLS statement in SAS (SAS Institute Inc., Cary, North Carolina) was used to conduct RRR analysis using iron intake as the response variable [6]. As there was only one response variable, one iron-related dietary pattern was extracted. IDP scores were calculated as the sum of the products of the factor loading coefficients and standardized daily intake of each food group associated with the pattern. We calculated a cumulative average IDP score at each time period to reduce variation within individuals and to represent long term habitual intake [37]. For example, the 1991 intake was used for the follow-up between 1991 and 1993, the average of the 1991 and 1993 intake was used for the follow-up between 1997 and 2000, and so on. Details on cumulative average IDP are illustrated on Additional file 1: Figure S1. In the sensitivity analysis, we also assessed the association between most recent IDP and cognitive function. In sensitivity analyses, we excluded 1991 and 1993 IDP as cognitive function test was not conducted in these surveys. As the main findings did not change, we decided to include 1991 and 1993 dietary data in our analysis.

#### Lead

Dietary lead of each participant was estimated based on the food intake described above and calculated using published food lead concentration data (mean lead in each food category) from Jiangsu Province (one of the nine provinces in CHNS) [23]. The lead concentration (μg/d) table was based on lead measurements in 2077 food samples from 23 food categories during 2007–2010.

### Covariates

Height, weight, and blood pressure were measured at each wave. Overweight/obesity was defined as BMI ≥24 kg/m^2^ [38]. Hypertension was defined as systolic blood pressure above 140 mmHg and/or diastolic blood pressure above 90 mmHg, or having known hypertension.

The following constructed sociodemographic variables were used: education (low: illiterate/primary school; medium: junior middle school, and high: high middle school or higher), per capita annual family income (recoded into tertiles as low, medium and high), urbanization levels [31] (recoded into tertiles as low, medium and high).

Physical activity level (Metabolic Equivalent of Task, (MET)) was estimated based on self-reported daily activities (including occupational, domestic, transportation, and leisure time physical activity) and duration using a Compendium of Physical Activities [39]. Smoking status was categorized into non-smokers, ex-smokers and current smokers. Alcohol drinking was categorized as yes or no. Self-reported diabetes and stroke were coded as yes or no.

### Statistical analysis

Cumulative mean IDP score was recoded into quartiles. The chi square test was used to compare differences between groups for categorical variables and ANOVA for continuous variables. We use mixed effect model in Stata to assess the association between IDP and cognitive function. A negative regression coefficient represents cognitive function decline. Four multivariable models were used: model 1 adjusted for age, gender and energy intake; model 2 further adjusted for intake of fat, smoking, alcohol drinking, physical activity, income, urbanization, and education; model 3 further adjusted for BMI and hypertension. The fourth model included lead/carbohydrate/iron intake in model 3. It was used to test whether the association between IDP and cognition was mediated by lead or carbohydrate intake by comparing the effect estimated before and after the adjustment of lead, carbohydrate or iron. We also excluded those with a global cognitive function score ≤ 4 and further adjusted for diabetes and stroke. The variables included in the multivariable models were known to be associated with cognition including socioeconomic status, lifestyle factors and chronic conditions. We chose these variables as covariates because they are both associated with food intake and importantly with cognitive function. Scatter plots were used to visually present the association between IDP and lead intake in 1997, 2000, 2004 and 2006 surveys.

To assess the association between cumulative IDP and the risk of poor cognitive function, we used mixed effect logistic regression adjusting for covariates the same as model 3 mentioned above. In sensitivity analyses, we also stratified our analysis by total meat intake (including pork, beef and poultry, above or below 50 g/d). To test the interaction between IDP and BMI, hypertension, meat intake and income on the association with poor cognition, a product term of each of the two variables was put in the regression model. All the analyses were performed using STATA 15.1 (Stata Corporation, College Station). Statistical significance was considered when *p* < 0.05 (two sided).

## Results

**Fig. 1:**
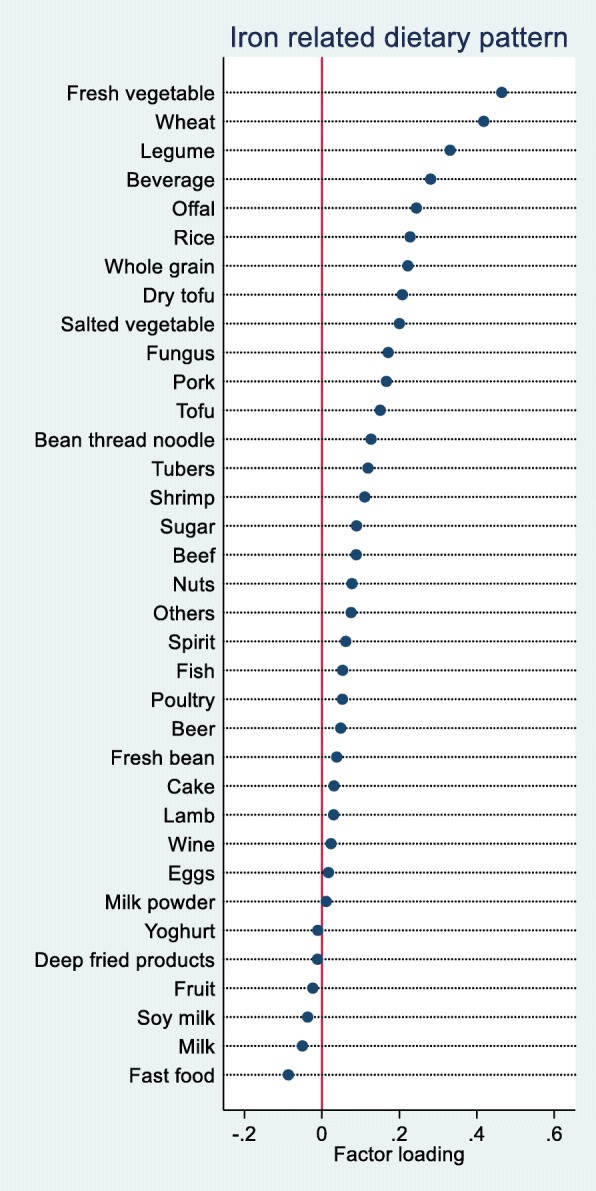
Factor loadings of iron related dietary pattern based on reduced rank regression

IDP derived using RRR methods with iron as a response variable was characterized by high loadings of fresh vegetable, wheat, legume, beverage (i.e. soft drinks, fruit juice and tea), offal, rice and whole grain (Fig. 1). The pattern explained 36.4% of the variation of iron intake. The intake of offal was low between 1991 and 2006 (mean 3.1 g/d, SD 15.4, more than 90% were non-consumers during the 3-day survey). The intake of rice, wheat, legume and fresh vegetable contributed 25.5, 25.0, 1.2 and 15.7% of the total iron intake, respectively.

**Fig. 2:**
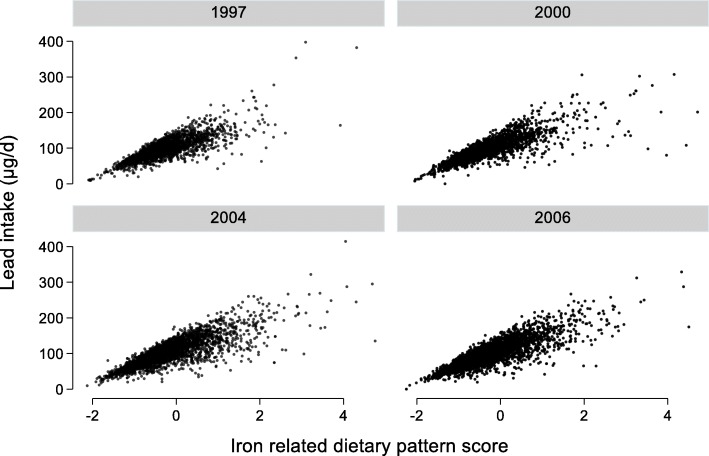
Association between iron-related dietary pattern and lead intake

Table 1 shows the sample characteristics among participants attending the first cognitive function test by quartiles of IDP. Across the quartiles of IDP, the intake of energy, protein, fat, carbohydrate, wheat, rice and fresh vegetable increased. However, there was no difference of fruit intake across quartiles of IDP. IDP was positively associated with smoking, alcohol drinking and physical activity level but inversely associated with age. The mean cumulative intake of iron in the high quartile of IDP was 31.0 mg (SD 12.2) as compared with 15.8 mg (SD 6.2) in the first quartile. IDP was significantly positively associated with lead intake (Fig. 2).

**Table 1:** Sample characteristics of Chinese adults aged ≥55 years old at the first cognitive function test by quartiles of iron related dietary pattern (*N* = 4685)

	Q1	Q2	Q3	Q4	*p*-value
	*N* = 1150	*N* = 1128	*N* = 1161	*N* = 1246	
Energy intake (kcal/d)	1625.8 (440.6)	2028.5 (1057.2)	2196.2 (552.1)	2516.4 (799.3)	< 0.001
Fat intake (g/d)	56.5 (30.3)	71.0 (112.6)	70.7 (36.2)	72.8 (58.6)	< 0.001
Protein intake (g/d)	47.1 (14.6)	60.0 (17.2)	67.1 (20.5)	78.4 (28.5)	< 0.001
Carbohydrate intake (g/d)	229.1 (69.9)	282.3 (80.1)	316.2 (88.9)	377.5 (121.5)	< 0.001
Most recent iron intake (mg/d)	13.8 (7.2)	18.1 (7.3)	21.2 (11.0)	26.8 (15.2)	< 0.001
Cumulative iron intake (mg/d)	15.8 (6.2)	21.2 (6.6)	24.5 (9.4)	31.0 (12.2)	< 0.001
Intake of fruit (g/d)	22.6 (69.4)	23.8 (73.8)	23.9 (83.9)	22.7 (90.2)	0.97
Intake of fresh vegetable (g/d)	183.8 (103.5)	254.4 (132.4)	298.4 (155.6)	352.3 (234.9)	< 0.001
Intake of rice (g/d)	190.9 (116.7)	232.1 (140.5)	252.9 (163.5)	237.7 (207.9)	< 0.001
Intake of wheat (g/d)	84.4 (90.9)	107.9 (110.8)	140.3 (138.3)	227.8 (226.3)	< 0.001
Intake of meat (g/d)	56.0 (58.1)	77.8 (74.4)	80.6 (83.4)	77.1 (102.3)	< 0.001
Age (years)	67.8 (9.0)	63.1 (7.3)	62.0 (6.8)	60.9 (6.1)	< 0.001
Sex					< 0.001
Men	356 (31.0%)	499 (44.2%)	594 (51.2%)	799 (64.1%)	
Women	794 (69.0%)	629 (55.8%)	567 (48.8%)	447 (35.9%)	
Education					0.002
Low	727 (77.5%)	735 (70.7%)	773 (71.8%)	832 (70.7%)	
Medium	105 (11.2%)	158 (15.2%)	163 (15.1%)	202 (17.2%)	
High	106 (11.3%)	146 (14.1%)	141 (13.1%)	142 (12.1%)	
Urbanization					< 0.001
Low	224 (19.5%)	218 (19.3%)	282 (24.3%)	468 (37.6%)	
Medium	274 (23.8%)	325 (28.8%)	368 (31.7%)	352 (28.3%)	
High	652 (56.7%)	585 (51.9%)	511 (44.0%)	426 (34.2%)	
Smoking					< 0.001
Non-smoker	879 (76.8%)	796 (70.7%)	765 (65.9%)	709 (57.0%)	
Ex-smokers	42 (3.7%)	33 (2.9%)	36 (3.1%)	66 (5.3%)	
Current smokers	224 (19.6%)	297 (26.4%)	359 (30.9%)	469 (37.7%)	
Survey year					< 0.001
1997	561 (48.8%)	537 (47.6%)	500 (43.1%)	514 (41.3%)	
2000	210 (18.3%)	171 (15.2%)	186 (16.0%)	207 (16.6%)	
2004	246 (21.4%)	239 (21.2%)	284 (24.5%)	324 (26.0%)	
2006	133 (11.6%)	181 (16.0%)	191 (16.5%)	201 (16.1%)	
Alcohol drinking (yes)	241 (21.4%)	305 (27.6%)	390 (34.0%)	498 (40.7%)	< 0.001
Physical activity (MET, hours/week)	58.1 (75.9)	87.2 (101.0)	91.3 (98.8)	111.9 (109.5)	< 0.001
BMI (kg/m^2^)	22.8 (3.8)	23.2 (3.7)	23.1 (3.5)	23.1 (3.4)	0.075
BMI ≥ 24 kg/m^2^	367 (34.9%)	408 (39.2%)	412 (37.9%)	407 (35.8%)	0.15
Hypertension (yes)	447 (41.2%)	375 (35.5%)	363 (32.8%)	375 (32.3%)	< 0.001
Diabetes (yes)	45 (4.0%)	36 (3.2%)	29 (2.6%)	39 (3.2%)	0.29
Stroke (yes)	34 (3.0%)	19 (1.7%)	18 (1.6%)	28 (2.3%)	0.082

Data are shown as n (%) or mean ± SD. *p* values were calculated from ANOVA or chi square test

The mean global cognition score was 12.1 (SD 6.8) in 1997. The prevalence of poor cognition ranged from 19.8 to 23.1% in the four waves of survey between 1997 and 2006. The annual cognitive function score decline was 0.1 (95% CI 0.07, 0.13).

IDP was related to cognitive function decline in a dose-response manner (Table 2). The difference in cognitive function between quartile 4 and quartile 1 of IDP was − 1.23 after adjusting for age, gender and energy intake. In the fully adjusted model, across quartiles of IDP, the regression coefficients (95% CI) were: 0, − 0.11 (− 0.50, 0.28), − 0.42 (− 0.84, 0.00), and − 0.79 (− 1.25, − 0.32), respectively. A similar positive association between IDP and memory decline was observed. The regression coefficients for memory across quartiles of IDP were: 0, − 0.15 (− 0.43, 0.13), − 0.31 (− 0.61, − 0.02), and − 0.37 (− 0.70, − 0.04), respectively. The associations between IDP and memory decline were attenuated and became statistically not significant after further adjustment of cumulative carbohydrate or iron intake. Similarly, the associations between IDP and memory decline were attenuated after adjustment for lead intake. This suggests the mediating effect of carbohydrates and lead in the relationship between IDP and cognition. Both carbohydrate and lead intake were inversely associated with cognitive function (data not shown).

**Table 2:** Regression coefficients (95% CI) for cognitive function by quartiles of iron related dietary pattern among Chinese adults aged ≥55 years old attending China Health and Nutrition Survey (*N* = 4852) between 1997 and 2006

	Dietary pattern quartiles				
	Q1 (low intake)	Q2	Q3	Q4 (high intake)	p for trend
*Global cognitive function*	Coef. (95% CI)				
Model 1^a^	0.00	−0.06 (−0.42–0.30)	**−0.53 (−0.92--0.15)**	**−1.23 (−1.65--0.81)**	< 0.001
Model 2^b^	0.00	0.00 (− 0.39–0.38)	− 0.32 (− 0.73–0.08)	**− 0.74 (−1.19--0.28)**	< 0.001
Model 3^c^	0.00	− 0.11 (− 0.50–0.28)	− 0.42 (− 0.84–0.00)	**− 0.79 (− 1.25--0.32)**	< 0.001
Model 3+ carbohydrate (quartiles)	0.00	0.15 (− 0.26–0.55)	0.05 (− 0.40–0.50)	− 0.19 (− 0.70–0.32)	0.373
Model 3 + lead (quartiles)	0.00	−0.01 (− 0.45–0.42)	−0.24 (− 0.74–0.27)	−0.57 (− 1.16–0.02)	0.035
Model 3 + iron (quartiles)	0.00	0.05 (−0.37–0.47)	−0.16 (− 0.63–0.31)	−0.41 (− 0.95–0.13)	0.080
Sensitivity analysis ^d^	0.00	0.01 (− 0.36–0.38)	−0.30 (− 0.69–0.09)	**−0.52 (− 0.96--0.09)**	0.006
*Verbal memory score*					
Model 1^a^	0.00	−0.13 (− 0.38–0.13)	**−0.44 (− 0.71--0.17)**	**−0.71 (− 1.00--0.41)**	< 0.001
Model 2^b^	0.00	−0.09 (− 0.37–0.18)	−0.25 (− 0.55–0.04)	**−0.33 (− 0.66--0.01)**	0.026
Model 3^c^	0.00	−0.15 (− 0.43–0.13)	−0.31 (− 0.61--0.02)	**−0.37 (− 0.70--0.04)**	0.020
Model 3+ carbohydrate (quartiles)	0.00	0.01 (−0.29–0.30)	− 0.07 (− 0.39–0.26)	−0.10 (− 0.47–0.26)	0.509
Model 3 + lead (quartiles)	0.00	−0.09 (− 0.41–0.22)	−0.17 (− 0.54–0.19)	−0.19 (− 0.61–0.24)	0.376
Model 3 + iron (quartiles)	0.00	−0.01 (− 0.31–0.29)	−0.09 (− 0.42–0.25)	−0.06 (− 0.45–0.33)	0.688
Sensitivity analysis^d^	0.00	−0.10 (− 0.38–0.17)	−0.23 (− 0.52–0.06)	−0.22 (− 0.55–0.10)	0.131

Regression coefficients and 95% CI were estimated with mixed effect regression models with different levels of adjustment

^a^ Model 1 adjusted for age, gender and energy intake

^b^ Model 2 further adjusted for intake of fat, smoking, alcohol drinking, income, urbanicity, education, and physical activity

^c^ Model 3 further adjusted for BMI and hypertension

^d^ Sensitivity analysis model 3 further adjusted for diabetes and stroke after excluding those with a global cognitive function score ≤ 4

All the adjusted variables are treated as time-varying covariates. Bold font represents *p*<0.05

In sensitivity analyses, the above association between IDP and cognition did not change after excluding those with a global cognitive function score below 4 or further adjusting for diabetes or stroke or lead intake.

However, no association between most recent IDP and cognition was found in multivariable mixed model (Additional file 1: Tables S1 and S2).

A borderline significant interaction (*p* = 0.085) between IDP and meat intake in relation to cognitive function was found (Table 3). The positive association between IDP and cognitive function decline was only observed among those with no or low intake of meat. Among those with meat intake < 50 g/d, there was a significant increase of odds ratio (OR) (95% CI) for global cognition score below 7 across quartiles of IDP: 1.00, 1.13 (0.83–1.53), 1.28 (0.93–1.76), and 1.93 (1.36–2.75), respectively. There were no significant interactions with urban-rural residence, overweight/obesity, hypertension, and gender.

**Table 3:** Odds ratio (95% CI) for global cognitive score below 7 across quartiles of iron related dietary pattern among Chinese adults aged ≥55 years old by characteristics, China Health and Nutrition Survey (*N* = 4852) between 1997 and 2006 ^a^

	Q1	Q2	Q3	Q4	p for interaction
	Coef. (95% CI)				
Overall sample	1.00	1.06 (0.86–1.30)	1.24 (0.99–1.54)	1.50 (1.17–1.93)	
Overweight/obesity					
No	1.00	1.07 (0.83–1.39)	1.26 (0.96–1.66)	1.54 (1.13–2.10)	0.997
Yes	1.00	1.06 (0.75–1.50)	1.26 (0.87–1.82)	1.53 (1.00–2.34)	
Hypertension					
No	1.00	1.06 (0.82–1.37)	1.21 (0.92–1.59)	1.59 (1.17–2.15)	0.766
Yes	1.00	1.01 (0.73–1.40)	1.27 (0.89–1.80)	1.34 (0.89–2.02)	
Income					
Low	1.00	1.24 (0.89–1.74)	1.24 (0.86–1.78)	1.32 (0.88–1.98)	0.330
Medium	1.00	0.79 (0.55–1.13)	1.03 (0.71–1.49)	1.42 (0.93–2.17)	
High	1.00	1.09 (0.73–1.62)	1.49 (0.96–2.31)	1.84 (1.12–3.02)	
Gender					
Men	1.00	1.27 (0.83–1.95)	1.59 (1.04–2.44)	2.10 (1.35–3.29)	0.497
Women	1.00	1.00 (0.78–1.27)	1.11 (0.85–1.45)	1.24 (0.90–1.70)	
Urbanization					
Low	1.00	1.52 (0.97–2.39)	1.24 (0.78–1.98)	1.94 (1.21–3.11)	0.323
Medium	1.00	0.95 (0.63–1.41)	1.25 (0.82–1.89)	1.29 (0.80–2.08)	
High	1.00	0.94 (0.70–1.25)	1.21 (0.88–1.67)	1.33 (0.89–1.98)	
Meat intake					
< 50 g/d	1.00	1.13 (0.83–1.53)	1.28 (0.93–1.76)	1.93 (1.36–2.75)	0.085
≥ 50 g/d	1.00	1.00 (0.75–1.32)	1.17 (0.86–1.59)	1.05 (0.73–1.52)	

^a^ Mixed effect logistic modes adjusted for age, gender, intake of energy and fat, smoking, alcohol drinking, income, urbanicity, education, and physical activity, BMI and hypertension. Stratification variables were not adjusted in the corresponding models

Income was categorized into low, medium and high based on tertiles of year specific income

When we limited the analyses to those who took the cognitive tests in at least two waves of survey, the findings remained unchanged. Intake of lead was positively associated with global cognition score < 7. However, adjusting for lead intake did not change the association between IDP and cognition.

In fully adjusted models, across the quartiles of IDP intake the OR (95% CI) for global cognition score < 7 were: 1.00, 1.06 (0.86–1.30), 1.24 (0.99–1.54), and 1.50 (1.17–1.93), respectively. The association became statistically insignificant after further adjustment of cumulative carbohydrate intake.

## Discussion

In this prospective cohort study of adults aged ≥55 years from CHNS, using RRR method we derived an iron-related dietary pattern characterized by a high intake of fresh vegetable, wheat, legumes, beverage, offal, rice and whole grain. A high intake of this predominantly plant-based IDP was associated with poor cognitive function. Meat consumption modified the association between IDP and cognition. The association between IDP and poor cognitive function was mainly seen among those with a low intake of meat but not those with a high intake of meat. The dietary pattern was also highly associated with lead, iron and carbohydrate intake. Our study suggests that the association between IDP and cognitive function may be mediated partly by iron, lead and carbohydrate intake in the Chinese population.

This is the first study using RRR method to construct dietary pattern with iron as an intermediate response variable. The dietary pattern approach used in the study helps to understand the complex nature of nutritional epidemiological studies. Our study shows that the single nutrient approach commonly used in the traditional epidemiological research can be confounded by many unmeasured factors. The current study of iron-related dietary pattern further confirms our previous findings on the association between iron intake and cognitive function [17]. The intake of iron in the fourth quartiles of IDP was 31 mg/d which is 2.5 times the recommended iron intake (i.e. 12 mg/d) for Chinese adults. Compared with the relationship between iron intake and poor cognition, the association between IDP and poor cognition showed a clearer dose response positive association. Specifically, across quartiles of iron intake, the OR for poor cognition were 1.00, 1.06 (0.87, 1.30), 1.09 (0.88, 1.35) and 1.30 (1.04, 1.64), respectively. The corresponding figures were 1.00, 1.06 (0.86–1.30), 1.24 (0.99–1.54), and 1.50 (1.17–1.93) across quartiles of iron related dietary pattern.

The focus on the link between iron intake and cognition can be dated back to 1956, when Harman hypothesized that free non-heme iron is a major contributor of neural and cognitive aging [40]. The evidence has been synthesized in several recent reviews showing the important role of iron in neurodegenerative diseases, as a redox-active ion that can cause oxidative stress in the cell [11, 41] Iron intake is related to iron deposits in the brain in animal studies [42], which may increase oxidative stress in the brain. In animal models, iron chelation has been shown to be effective in treating neurodegenerative diseases, such as Parkinson’s and Alzheimer’s diseases [41].

Some components of our IDP (fresh vegetables, legumes and whole grain) were similar with Mediterranean diet. However, our IDP also differ from the Mediterranean diet as it does not have high loadings of fish and nuts, which have showed beneficial role for cognition function [43, 44]. Overall, the beneficial effects of Mediterranean diet on cognition has been well documented [7]. Traditional Chinese diet has similarity to the Mediterranean diet as shown by its high intake of vegetable, whole grains, and vegetable oil. In CHNS, Qin et al found that those with a high adapted Mediterranean diet score were associated with a slower cognitive decline [45]. The main contributors of the observed association were fish, fruits and lower intake of animal-source cooking fats from the aforementioned Mediterranean diet. However, the intake of vegetable, legume, fiber-rich grains showed no benefits on cognition. In Hong Kong, no association between Mediterranean diet and cognition was found [46].

The interaction between IDP and meat consumption is intriguing. The positive association between IDP and poor cognition was only found among those with no or a low meat intake, suggesting the importance of a balanced diet. This finding is also supported by the current knowledge on the importance of protein-rich diet for peoples’ health in addition to plant/vegetable consumption. Previous studies have shown the beneficial role of the higher-protein diets, which improve adiposity, blood pressure and triglyceride levels, which are in turn related to cognitive impairment disorders such as Alzheimer’s and Parkinson’s [47]. There is growing interest in the association between a protein-rich diet and cognition in the epidemiology studies. For example, our findings are consistent with another study using CHNS data. Xu et al found that protein-rich dietary pattern (high intake of milk, eggs and soymilk) was positively but a starch-rich dietary pattern (high intake of salted vegetable, whole grain and legumes) was inversely associated with global cognition in CHNS [48].

High carbohydrate intake was associated with poor cognition in our study and explained the association between IDP and cognition. This finding is supported by several studies. For example, a low glycemic index (GI) breakfast has been shown to be in favour of cognitive performance later in the morning among adults [49]. Low carbohydrate diet has been shown to be beneficial for cognition function among older adults with mild cognitive impairment [19]. Among diabetic patients, a low-GI carbohydrate meal is associated with better cognitive performance than a high GI meal [21].

Food heavy metal contamination (e.g. lead, cadmium and arsenic) has been a greater public concern in China due to environmental pollution [50]. The mean dietary lead intake in the Chinese population was estimated to be 73.9 μg/d [25]. In the current study, IDP was highly correlated with lead intake. Lead intake is a known risk factor for poor cognition. It may explain the association between IDP and poor cognition. The positive association between IDP and cognition may suggest a collective effect of high iron and heavy metals. As IDP was inversely associated with CRP in 2009 (data not shown), the association between IDP is unlikely to be mediated by inflammation.

Our study has several limitations. Wheat contains twice the amount of iron than rice and contributes mainly to the IDP. As there is a large geographic variation on wheat consumption in China, our findings may be confounded by unmeasured factors related to regions. The use of a regional food lead concentration table is another limitation as the contamination level is likely to be varied by regions. However, the estimation of lead intake only serves the purpose to explore the possible explanation of the link between IDP and cognition. Under-reporting of diabetes is another limitation. We were unable to test whether diabetes mediates the association between IDP and cognition. Furthermore, we do not have occupational information, which has been linked to cognitive impairment. Nevertheless, education levels and physical activities were accounted for. The strength of the study is the repeated measure of dietary intake over a long period of time. We are able to adjust for several confounding factors.

## Conclusion

Iron related diet may increase the risk of poor cognition. This link is particularly strong for individuals with no or low meat consumption, underscoring the importance of a balanced diet. The link may be partly explained by a high intake of lead, iron and carbohydrate. As the burden of obesity and other non-communicable diseases increase, people are increasingly seeking a plant-based diet to manage body weight. Intake of adequate animal food is needed to prevent cognition decline among those with a high intake of plant-based diet. The role of iron and heavy metal contamination on cognition needs further investigation.

## Additional file

Additional file 1: **Figure S1.** Sample flowchart of participants attending China Health and Nutrition Survey. **Table S1.** Regression coefficients (95% CI) for cognitive function by quartiles of recent iron related dietary pattern among Chinese adults aged ≥55 years old attending China Health and Nutrition Survey (*N* = 4852) between 1997 and 2006. **Table S2.** Odds ratio (95% CI) for global cognitive score below 7 across quartiles of recent iron related dietary pattern among Chinese adults aged ≥55 years old by characteristics, China Health and Nutrition Survey (N = 4852) between 1997 and 2006. (DOCX 50 kb)

## Data Availability

The study used open access data. The original data are available from website: https://www.cpc.unc.edu/projects/china
