# Supplementary material for: Iron-related dietary pattern increases the risk of poor cognition
Source: Nutr J. 2019 Aug 29;18:48. doi: 10.1186/s12937-019-0476-9 (PMC6716885; doi:10.1186/s12937-019-0476-9)
Supplement: Supplementary file 1 — Figure S1. Sample flowchart of participants attending China Health and Nutrition Survey. Table S1. Regression coefficients (95% CI) for cognitive function by quartiles of recent iron related dietary pattern among Chinese adults aged ≥55 years old attending China Health and Nutrition Survey (N = 4852) between 1997 and 2006. Table S2. Odds ratio (95% CI) for global cognitive score below 7 across quartiles of recent iron related dietary pattern among Chinese adults aged ≥55 years old by characteristics, China Health and Nutrition Survey (N = 4852) between 1997 and 2006. (DOCX 50 kb) [file 12937_2019_476_MOESM1_ESM.docx]

**Figure S1** Sample flowchart of participants attending China Health and Nutrition Survey

**
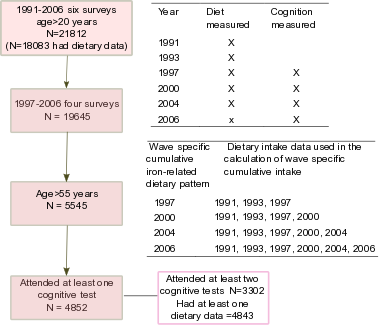
**

**Table S1** Regression coefficients (95% CI) for cognitive function by quartiles of recent iron related dietary pattern among Chinese adults aged ≥ 55 years old attending China Health and Nutrition Survey (N=4852) between 1997 and 2006

|  |  | Dietary pattern quartiles |  |  |  |
| --- | --- | --- | --- | --- | --- |
|  | Q1 (low intake) | Q2 | Q3 | Q4 (high intake) | p for trend |
| *Global cognitive function* | Coef. (95% CI) | | | |  |
| Model 1 ^a^ | 0.00 | 0.67(0.34-1.00) | 0.39(0.04-0.74) | 0.46(0.05-0.86) | 0.12 |
| Model 2 ^b^ | 0.00 | 0.41(0.05-0.78) | 0.27(-0.12-0.66) | 0.23(-0.24-0.70) | 0.519 |
| Model 3 ^c^ | 0.00 | 0.35(-0.02-0.73) | 0.22(-0.18-0.63) | 0.14(-0.35-0.62) | 0.764 |
| Model 3+ carbohydrate (quartiles) | 0.00 | 0.41(0.03-0.79) | 0.34(-0.07-0.74) | 0.18(-0.30-0.67) | 0.57 |
| Model 3+lead (quartiles) | 0.00 | 0.44(0.06-0.83) | 0.39(-0.03-0.81) | 0.37(-0.13-0.88) | 0.224 |
| Model 3+iron (quartiles) | 0.00 | 0.42(0.04-0.80) | 0.36(-0.05-0.77) | 0.34(-0.15-0.83) | 0.26 |
| Sensitivity analysis ^d^ | 0.00 | 0.45(0.08-0.82) | 0.23(-0.16-0.62) | 0.24(-0.22-0.71) | 0.568 |
| *Verbal memory score* |  |  |  |  |  |
| Model 1 ^a^ | 0.00 | 0.40(0.16-0.64) | 0.20(-0.05-0.46) | 0.18(-0.11-0.47) | 0.523 |
| Model 2 ^b^ | 0.00 | 0.27(0.00-0.53) | 0.17(-0.12-0.45) | 0.08(-0.26-0.42) | 0.818 |
| Model 3 ^c^ | 0.00 | 0.24(-0.03-0.51) | 0.15(-0.15-0.44) | 0.05(-0.30-0.40) | 0.947 |
| Model 3+ carbohydrate (quartiles) | 0.00 | 0.28(0.00-0.55) | 0.22(-0.07-0.51) | 0.08(-0.27-0.43) | 0.784 |
| Model 3+lead (quartiles) | 0.00 | 0.29(0.02-0.57) | 0.26(-0.05-0.56) | 0.19(-0.17-0.56) | 0.397 |
| Model 3+iron (quartiles) | 0.00 | 0.29(0.02-0.57) | 0.25(-0.05-0.55) | 0.20(-0.16-0.55) | 0.372 |
| Sensitivity analysis ^d^ | 0.00 | 0.29(0.01-0.56) | 0.20(-0.09-0.50) | 0.22(-0.12-0.57) | 0.333 |

Regression coefficients and 95% CI were estimated with mixed effect regression models with different levels of adjustment.

^a^ Model 1 adjusted for age, gender and energy intake.

^b^ Model 2 further adjusted for intake of fat, smoking, alcohol drinking, income, urbanicity, education, and physical activity.

^c^ Model 3 further adjusted for BMI and hypertension.

^d^ Sensitivity analysis model 3 further adjusted for diabetes and stroke after excluding those with a global cognitive function score ≤4.

All the adjusted variables are treated as time-varying covariates.

**Table S2** Odds ratio (95% CI) for global cognitive score below 7 across quartiles of recent iron related dietary pattern among Chinese adults aged ≥55 years old by characteristics, China Health and Nutrition Survey (N=4852) between 1997 and 2006 ^a^

|  | Q1 | Q2 | Q3 | Q4 | p for interaction |
| --- | --- | --- | --- | --- | --- |
|  |  |  | Coef. (95% CI) |  |  |
| Overall sample | 1.00 | 0.94(0.77-1.15) | 0.94(0.75-1.17) | 1.08(0.83-1.42) |  |
| Overweight/obesity |  |  |  |  |  |
| No | 1.00 | 0.96(0.75-1.22) | 0.94(0.72-1.24) | 1.12(0.80-1.57) | 0.919 |
| Yes | 1.00 | 0.99(0.70-1.41) | 1.00(0.69-1.46) | 1.11(0.70-1.76) |  |
| Hypertension |  |  |  |  |  |
| No | 1.00 | 0.90(0.70-1.15) | 0.88(0.67-1.15) | 0.90(0.64-1.26) | 0.437 |
| Yes | 1.00 | 1.03(0.74-1.43) | 1.02(0.71-1.46) | 1.46(0.95-2.23) |  |
| Income |  |  |  |  |  |
| Low | 1.00 | 0.75(0.54-1.04) | 0.78(0.54-1.11) | 0.99(0.63-1.54) | 0.516 |
| Medium | 1.00 | 1.00(0.71-1.41) | 0.94(0.64-1.40) | 1.05(0.65-1.70) |  |
| High | 1.00 | 1.30(0.86-1.97) | 1.24(0.79-1.96) | 1.25(0.73-2.13) |  |
| Gender |  |  |  |  |  |
| Men | 1.00 | 1.03(0.70-1.50) | 1.11(0.74-1.64) | 1.54(0.97-2.43) | 0.705 |
| Women | 1.00 | 0.89(0.70-1.13) | 0.85(0.64-1.11) | 0.88(0.62-1.23) |  |
| Urbanization |  |  |  |  |  |
| Low | 1.00 | 0.67(0.44-1.02) | 0.88(0.57-1.36) | 0.78(0.45-1.36) | 0.135 |
| Medium | 1.00 | 0.92(0.63-1.35) | 1.06(0.71-1.61) | 1.49(0.92-2.43) |  |
| High | 1.00 | 1.10(0.82-1.47) | 0.83(0.59-1.16) | 0.95(0.63-1.45) |  |
| Meat intake |  |  |  |  |  |
| <50 g/d | 1.00 | 0.83(0.62-1.11) | 1.18(0.86-1.62) | 1.45(0.99-2.13) | 0.005 |
| ≥50 g/d | 1.00 | 1.04(0.79-1.37) | 0.73(0.54-1.01) | 0.78(0.53-1.15) |  |

^a^ Mixed effect logistic modes adjusted for age, gender, intake of energy and fat, smoking, alcohol drinking, income, urbanicity, education, and physical activity, BMI and hypertension. Stratification variables were not adjusted in the corresponding models.

Income was categorized into low, medium and high based on tertiles of year specific income.
